# An Unusual Presentation of Cogan’s Syndrome With a Renal Tumor: A Report of a Rare Case

**DOI:** 10.7759/cureus.42123

**Published:** 2023-07-19

**Authors:** John P Kirsch, Madeline M Nottoli, Dawood Findakly, Jue Wang

**Affiliations:** 1 Internal Medicine, Creighton University School of Medicine, Phoenix, USA; 2 Hematology and Oncology, Louisiana State University Health Shreveport, Shreveport, USA; 3 Genitourinary Oncology, Dignity Health Cancer Institute, St. Joseph’s Hospital and Medical Center, Phoenix, USA; 4 Genitourinary Oncology, Creighton University School of Medicine, St. Joseph’s Hospital and Medical Center, Phoenix, USA

**Keywords:** cogan’s syndrome, interstitial keratitis, aortitis, renal cell carcinoma, third-degree av block

## Abstract

Cogan’s syndrome (CS) is a rare disorder of an unknown origin characterized by inflammatory eye disease and vestibuloauditory symptoms, primarily affecting young white adults, without a hereditary pattern. The exclusion of other diseases makes diagnosis difficult, and it is likely underreported in the literature. A 74-year-old previously healthy African American male presented with ear and jaw pain, later accompanied by vestibular symptoms, fever of unknown origin, aortitis, and a third-degree heart block. The workup revealed incidental renal cell carcinoma and interstitial keratitis. This case highlights the challenge of diagnosing an atypical presentation of CS with late-onset interstitial keratitis by excluding other complex syndromes.

## Introduction

Cogan’s syndrome (CS) is a rare autoimmune disease of unknown origin characterized primarily by vestibuloauditory and inflammatory eye symptoms [1]. It was first described in 1945 by Dr. David Cogan as, “a syndrome of non-syphilitic interstitial keratitis and vestibuloauditory symptoms” [2,3]. Since then, only a few hundred cases have been reported in the literature [4].

CS primarily affects young, Caucasian adults in their third to fourth decade of life [5]. While the etiology is unknown, it is thought to arise from environmental infectious triggers such as upper respiratory tract infections [5]. There are two forms of CS that differ by the timeline of symptom onset. Typical, or classical, CS is diagnosed when vestibuloauditory and ocular symptoms arise within two years of one another. Atypical CS is diagnosed when there is a delay of two or more years between the onset of vestibuloauditory and ocular symptoms [1]. Vestibuloauditory symptoms primarily consist of tinnitus, vertigo, ear fullness, and pressure. CS is a cause of progressive deafness. It is generally assumed that it is an autoimmune disease. Autoimmune inner ear diseases are unusual causes of progressive bilateral sensorineural hearing loss and vestibular defect. Patients may also suffer from nausea, vomiting, nystagmus, and ataxia [6,7]. Ocular symptoms consist primarily of interstitial keratitis, resulting in eye sensitivity, redness, and blurred vision. Other ocular symptoms may include conjunctivitis, uveitis, scleritis, optic neuritis, and glaucoma [1]. Additionally, up to 70% of patients with CS experience systemic symptoms, such as fevers, weight loss, myalgias, headaches, rash, and fatigue [8].

Much heterogeneity exists in the limited literature on CS, as each patient’s onset, severity, and assortment of symptoms may vary. Considering the variable onset and development of symptoms, as well as the lack of specific laboratory tests, the diagnosis of CS is a challenge and is often based on a good response to corticosteroid treatment [8].

We present a peculiar case of a typical (classical) presentation of CS in a previously healthy 74-year-old African American man.

## Case presentation

A 74-year-old African American male was in his usual state of health until November 2021, when he developed left ear pain and left jaw pain. His primary care physician diagnosed him with otitis media, and he completed 14 days of amoxicillin with no improvement. Shortly after he developed worsening headache, lightheadedness, decreased left-sided hearing, and generalized weakness. He was then started on oral azithromycin and antibiotic ear drops which led to no improvement.

One month later, he was evaluated by otolaryngology. He was found to have a left ear effusion and underwent tympanostomy tube placement. The following month, the patient experienced worsening headache, lightheadedness, and ear pain following a plane flight, and was initiated on oral steroid therapy. He reported no improvement and increased weakness and presented to the emergency room (ER). Computed tomography (CT) of the head showed no acute intracranial abnormality, and he was treated for pain control.

Follow-up with otolaryngology revealed no abnormalities post-tympanostomy tube placement. However, the patient deteriorated over the following days with increasing weakness and ear pain, and he returned to the ER where he was again treated for pain control.

**Figure 1:**
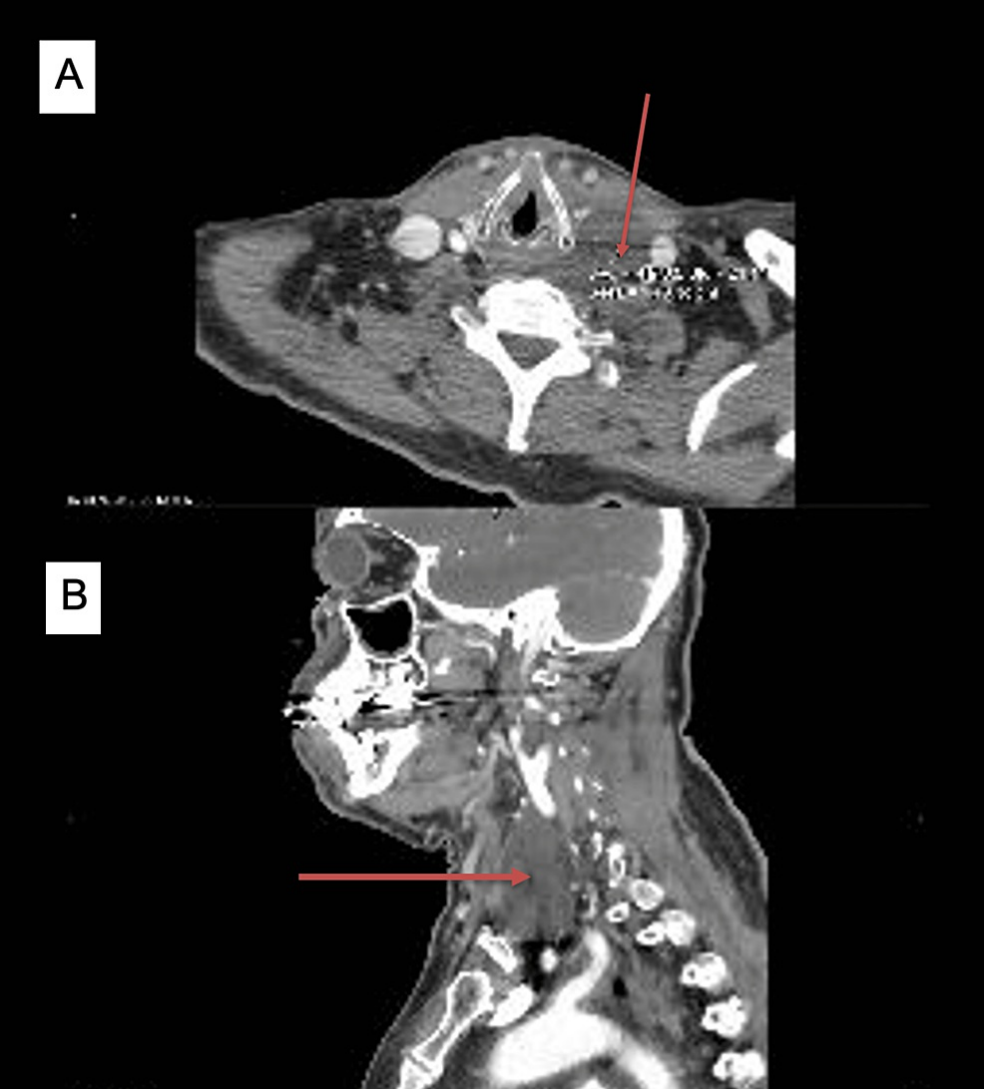
CT cervical soft tissue showing 5 cm L periesophageal/supraclavicular base fluid collection most consistent with lymphocele versus foregut duplication cyst. A: transverse section; B: sagittal section. CT = computed tomography

When seen by neurology the following week, blood work was drawn which revealed leukocytosis. Overnight, the patient’s condition worsened again, and he returned to the ER for the third time in one week with a chief complaint of generalized weakness. He was also found to have a temperature of 102.3°F. He then underwent an extensive workup (Table 1). His COVID-19 test and respiratory panel were both negative, and his workup including HIV and syphilis testing was unremarkable. CT of the cervical soft tissue with contrast revealed a 5 cm left paraesophageal/supraclavicular base fluid collection most consistent with lymphocele versus foregut duplication cyst (Figure 1).

**Table 1:** Pertinent imaging and associated findings. CT = computed tomography; MRI = magnetic resonance imaging; DVT = deep vein thrombosis

Imaging site and modality	Results
CT cervical soft tissue with contrast	Fluid collection in L prevertebral space from the level of C2 to the thoracic inlet, extending toward the left side, displacing the left-sided gland anteriorly
CT cervical soft tissue with contrast	5 cm L paraesophageal/supraclavicular base fluid collection most consistent with lymphocele vs. foregut duplication cyst
MRI cervical spine	Degenerative changes of the cervical spine, no discrete disc herniation, and no pathological enhancement. The cervical spinal cord showed normal enhancement. Fluid collection in L anterior neck, C5-at least T1 vertebral body
MRI thoracic spine	No acute findings
MRI lumbar spine	No acute findings
CT angiogram	Mild stenosis of the right vertebral artery due to atherosclerotic disease corroborated the cystic lesion within the L neck base
MRI brain	No acute findings
Lumbar puncture	Clear fluid. No acute findings
Bilateral lower extremity ultrasound	No DVT

The patient was subsequently admitted under internal medicine for pyrexia of unknown origin. Laboratory studies showed continued leukocytosis and further history revealed a weight loss of 10 kg over a two-month period. Initial labs on admission showed normocytic anemia, leukocytosis, thrombocytosis, and hypoalbuminemia (Table 2).

**Table 2:** Pertinent laboratory results. MCV = mean corpuscular volume; Hbg = hemoglobin; WBC = white blood count; ESR = erythrocyte sedimentation rate; CRP = C-reactive protein

Laboratory parameter	Reference range	Value
MCV (fL)	80–100	84
Hgb (g/dL)	13.5–17.5	8.7
WBC (thousand/µL)	4,500–11,000	20,400
Neutrophils (%)	54–62	83.6
Platelets (thousand/µL)	150,000–400,000	428,000
Albumin (g/dL)	3.5–5.5	2.4
ESR (mm/hour)	0–15	>130
CRP	<10	>400

After evaluation by otolaryngology, the patient underwent left neck dissection with direct laryngoscopy and bronchoscopy a few days later which were negative for malignancy, which had been suspected given the high erythrocyte sedimentation rate (ESR) and unexplained weight loss (Table 3).

**Table 3:** Pathology findings of arytenoid biopsy and left neck dissection.

Site	Pathology findings
Right arytenoid, biopsy	Squamous mucosa with focal acute inflammation. Negative for dysplasia or malignancy
Left neck dissection contents, excision	Consistent with cystic parathyroid. Negative for malignancy

The patient was treated with IV antibiotics, including vancomycin and cefepime but had no clinical improvement.

**Figure 2:**
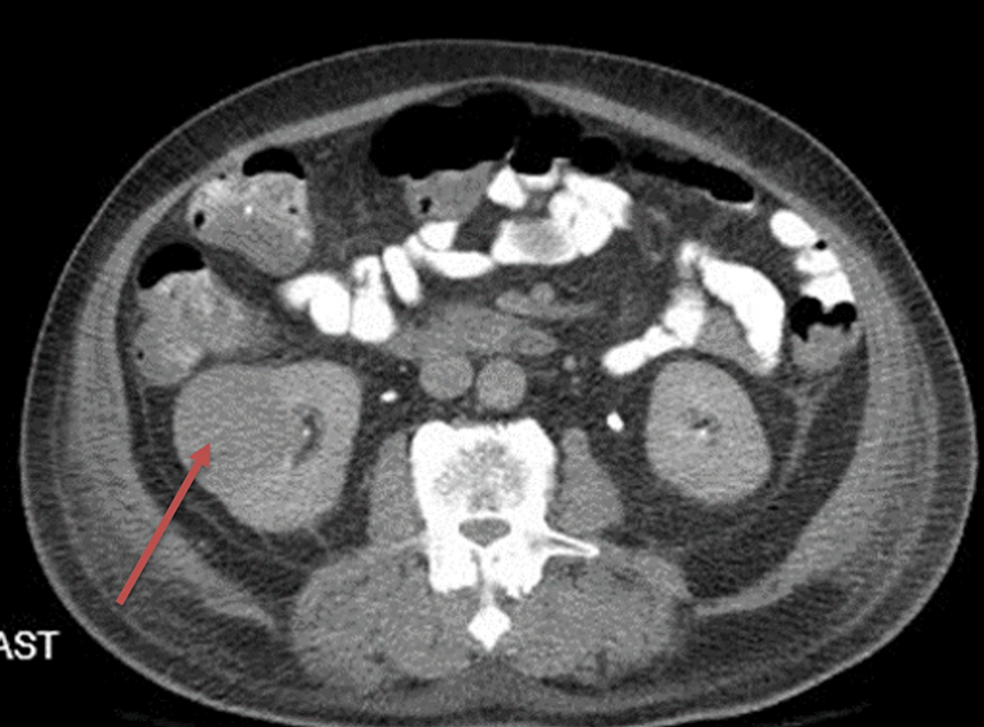
A large 5.5 × 4.4 cm exophytic hypervascular right renal mass (red arrow), transverse view.

Four days later, the patient underwent a CT scan of the chest, abdomen, and pelvis, which showed an incidental 5.5 × 4.4 cm exophytic, hypervascular right renal mass concerning for renal cell carcinoma (Figure 2). Genitourinary oncology was consulted for this finding and ordered inflammatory markers, including ESR, C-reactive protein (CRP), and interleukin-6 (IL-6). The patient’s inflammatory markers were elevated with an ESR of >130 mm/hour and a CRP of >400 mg/dL (Table 2). Infectious disease workup showed no evidence of tuberculosis, varices, bacterial, fungal, or atypical infection. On assessment with genitourinary oncology, the patient did not appear septic during intervals of intermittent fever break, and the differential diagnoses of autoimmune vasculitis and paraneoplastic leukocytosis were discussed.

Because paraneoplastic leukocytosis is a diagnosis of exclusion, rheumatology was consulted to investigate the differential diagnosis of vasculitis, as well as connective tissue diseases such as systemic lupus erythematosus, sarcoidosis, and granulomatosis with polyangiitis.

A temporal artery biopsy showed a medium-sized artery without significant inflammation. Elastin van Gieson staining demonstrated preserved internal elastic lamina.

The patient was seen by urology and underwent a partial nephrectomy four weeks later which yielded a low-grade neoplasm (Table 4).

**Table 4:** Pathology findings of the renal mass obtained after partial nephrectomy.

Site	Pathology findings
Kidney, right, lateral margin, excision	Renal parenchyma negative for malignancy
Kidney, right, deep margin, excision	Renal parenchyma negative for malignancy
Kidney, right, mass, right partial nephrectomy	Low-grade oncocytic renal neoplasm (5.6 cm), limited to kidney, margins negative for neoplasm
Fat over the renal mass, excision	Mature adipose tissue negative for neoplasm

According to the pathology report, the specimen demonstrated characteristics of a low-grade oncocytic renal neoplasm. The differential diagnosis, based on histology, included a low-grade oncocytoma with atypical features or a low-grade renal cell carcinoma, the unclassified type, with oncocytoma-like features. Given the low Ki-67 proliferative index, an indolent clinical behavior was likely. Although a definitive cancer diagnosis was uncertain, the pathologic stage for an unclassified renal cell carcinoma would be pT1b.

The patient was subsequently discharged home, followed up with genitourinary oncology, and was readmitted to the hospital two weeks later for recurrent fever and leukocytosis. He was admitted and evaluated by a multidisciplinary team, including infectious disease, oncology, rheumatology, interventional radiology, and cardiology.

Four days into his admission, the patient was incidentally found to have evidence of a complete heart block on telemetry but denied any symptoms of shortness of breath, lightheadedness, or chest pain. An echocardiogram was negative for signs of structural disease. The patient denied any previous cardiovascular issues aside from a diagnosis of hypertension. The patient’s third-degree heart block was evaluated by cardiology and electrophysiology, and no etiology for his heart block was established.

The patient’s blood and urine cultures were negative for viruses, atypical bacteria, and spirochete infections. Further infectious disease workup yielded negative results for hepatitis B surface antigen, hepatitis C, cytomegalovirus, West Nile virus, *Borrelia burgdorferi*, histoplasmosis, toxoplasmosis, and brucellosis. Immunology workup was negative for antinuclear antibody, antiextractable nuclear antigen/centromere, and notably, antineutrophil cytoplasmic antibody. No paraprotein was detected and immunoglobulin levels were within normal limits. Complete blood count with differential showed continued leukocytosis with neutrophilia, marked normocytic anemia, and moderate thrombocytosis. One week later, the patient underwent a bone marrow biopsy (Table 5).

**Table 5:** Results from bone marrow aspirate smears, clot section, and core biopsy.

Site/Modality	Result
Aspirate smear	Maturing trilineage hematopoiesis with megakaryocytic hyperplasia. Increased iron stores, 3+
Bone marrow core	30% cellular bone marrow. Moderate polytypic plasmacytosis
Flow cytometric analysis	No evidence of increased blasts, frank dysplasia, or lymphoid neoplasm

The patient was also evaluated by gastroenterology (GI) due to concern for possible gastrointestinal bleeding. Biopsies of the esophagus revealed *Candida esophagitis* but no evidence of dysplasia or malignancy.

A repeat CT of the chest, abdomen, and pelvis showed post-partial nephrectomy change, as well as diffuse circumferential aortic inflammation, which was reported to represent aortitis versus less likely retroperitoneal fibrosis.

Antibiotics were discontinued because there was no evidence of infection, and the patient was subsequently started on antifungal treatment for esophageal candidiasis. He was discharged on a high-dose steroid taper for aortitis/vasculitis by rheumatology. He also followed up with otolaryngology for hearing loss.

Two weeks later, the patient followed up with both urology and medical oncology, where laboratory studies showed acute renal failure, hyperglycemia, and hyponatremia. The patient was subsequently admitted to the hospital for hyperglycemia secondary to steroid use. Blood glucose was found to be over 700 mg/dL. Several days later, the patient was discharged home with insulin, and shortly after returned to oncology for a follow-up.

One month later, the patient underwent an internal auditory canal magnetic resonance imaging for continued lightheadedness and dizziness. The results displayed enhancement of the right membranous labyrinth, a finding with lower specificity that may be seen in CS.

The following month, the patient presented to the ER with a chief complaint of left eye redness. Upon further questioning, he noted additional pain, vision loss, and headache. He was diagnosed with interstitial keratitis, another finding of CS, and was discharged on prednisone and topical Tobramycin.

To date, the patient’s hearing loss has been stable since the initial evaluation. The patient was unable to tolerate prednisone and was switched to methotrexate. Clinically, he has shown significant improvement with resolved fever and lives independently with minor residual symptoms. The patient’s inflammatory lab values have also been reflective of his improvement since the initiation of treatment (Table 6).

**Table 6:** Inflammatory laboratory values before and after steroid medication. WBC = white blood count; ESR = erythrocyte sedimentation rate; CRP = C-reactive protein; IL-6 = interleukin 6

Laboratory parameter	Reference range	Values	
		Prior to steroids	After steroids
WBC	4500–11,000/mm^3^	15.9 (2/28/22)	6.1 (12/26/22)
ESR	0–15 mm/hour	79 (2/28/22)	20 (7/18/22)
CRP	<10.0	123.7 (2/28/22)	0.8 (8/22/22)
IL-6	<2.0	48.9 (2/7/22)	<2.0 (8/9/22)

## Discussion

This patient’s extensive disease course, while complicated, eventually revealed a fairly clear diagnosis of CS. His presentation included vestibular symptoms with hearing loss and tinnitus, sinusitis, otitis media, fevers, leukocytosis, weight loss, arthralgias, anemia, third-degree heart block, and a (likely unrelated) renal mass. This patient also displayed the commonly associated finding of aortitis, as well as late-onset interstitial keratitis [7,9,10].

Features of this case that are unusual include the age and ethnicity of the patient. CS has been shown to largely affect young Caucasian patients, whereas our patient was an older African American gentleman. While it is possible that he has unknowingly been affected by this condition for years, it is uncommon to be diagnosed at this age, and he did not suffer from any symptoms as a young adult. Notably, the patient initially lacked the most typical ocular symptom of non-syphilitic keratitis, which is a hallmark for patients with this condition [11,12]. He did, however, report some ocular symptoms over the prior three years and ultimately did develop interstitial keratitis, albeit months after the onset of his other symptoms. Despite the patient’s unusual clinical presentation, Classical CS is the best-fitting diagnosis, as the onset of interstitial keratitis did occur within two years of vestibular symptoms [13]. Additionally, while the atrioventricular block is not a novel association with this syndrome, it has only been documented once before in the literature as of 2005, further contributing to the unorthodox nature of this patient’s disease course [14].

Another point worth mentioning is the importance of thorough imaging. It could be argued that a more extensive initial imaging series, including a CT scan of the abdomen and pelvis, would have been indicated after the diagnosis of a neck mass. If that would have been the case, the renal tumor would have been diagnosed earlier, although it likely would not have made a difference in the timeline of his CS diagnosis.

## Conclusions

As the disease course unfolded, the CS criteria for vestibular, ocular, and aortic clinical presentations were eventually met, but in a metachronous manner. The diagnosis of rare diseases requires meticulous attention to the clinical course as well as the ability to view the case in the context of a larger picture despite the existence of many small details and other possible diagnoses such as a renal tumor. This case represents a challenge many clinicians face in daily practice and serves as a reminder that multiple unrelated diagnoses may sometimes provide the best explanation for a complex presentation.
